# Physiological and Biochemical Indicators of Urban Environmental Stress in *Tilia*, *Celtis*, and *Platanus*: A Functional Trait-Based Approach

**DOI:** 10.3390/plants14223451

**Published:** 2025-11-11

**Authors:** Danijela Arsenov, Milan Borišev, Nataša Nikolić, Rita Horak, Slobodanka Pajević

**Affiliations:** 1 Department of Biology and Ecology, Faculty of Sciences, University of Novi Sad, 21000 Novi Sad, Serbia; milan.borisev@dbe.uns.ac.rs (M.B.); natasa.nikolic@dbe.uns.ac.rs (N.N.); slobodanka.pajevic@dbe.uns.ac.rs (S.P.); 2 Hungarian Language Teacher Training Faculty, University of Novi Sad, 24000 Subotica, Serbia; horakrita83@gmail.com

**Keywords:** photosynthetic performances, climate acclimatization, urban resilience, oxidative stress biomarkers, tree vitality, stress management strategies, urban greening

## Abstract

Urban trees are exposed to multiple co-occurring stressors, including heat, drought, and pollution driven by intensified urbanization and climate change. These environmental pressures can compromise tree vitality by disrupting photosynthetic performance and oxidative balance. In this study, we assessed the structural, physiological, and biochemical responses of three common urban tree species (*Tilia platyphyllos*, *Celtis occidentalis*, and *Platanus × hispanica*) growing under urban environmental conditions in Novi Sad, Serbia. Leaf traits were measured during June and August to capture seasonal stress variation. Structural indicators (SPAD, leaf thickness, leaf temperature differential), chlorophyll fluorescence traits (*Φ*_II_, *Φ*_NPQ_, *F*_v′_/*F*_m′_), oxidative stress biomarkers (TBARS, proline, GSH), and antioxidant enzyme activities (APX, CAT) were quantified. The Tree Health Risk Index (THRI) was calculated to integrate multilevel responses. Results revealed species-specific differences, with *Tilia* exhibiting the highest sensitivity, characterized by notable photochemical declines and oxidative stress under urban conditions. *Celtis* showed moderate resilience, while *Platanus* demonstrated the most robust performance and emerged as a promising candidate for climate-resilient urban sites. Heatmap clustering and trait contribution analyses confirm oxidative stress biomarkers and chlorophyll fluorescence traits as key indicators of urban stress. This study emphasizes the importance of integrating functional trait-based approaches for assessing tree health in urban greening.

## 1. Introduction

Global climate change is an undeniable reality, with projections indicating an average temperature increase of 1.1–5.7 °C over the next century, creating a global concern [1]. This trend is exacerbated by urbanization, which intensifies local stress conditions through the urban heat island effect, reduced soil moisture, and elevated air pollution levels, significantly impacting cities’ ecosystems and environments [2,3]. Urban environments thus impose a complex combination of abiotic stressors, including heatwaves, drought, and loss of soil quality, that act simultaneously and interactively, forming a complex network of environmental drivers that can disrupt key metabolic pathways in plants [4]. In urban settings, climate-induced stressors are often exacerbated by anthropogenic pressures, particularly due to the ongoing increase in vehicular emissions. These emissions release substantial quantities of organic and inorganic pollutants, including heavy metals, particulate matter, volatile organic compounds, and harmful gases, which collectively provoke multiple adverse effects on plant growth, development, metabolism, and overall survival [5,6,7].

Roadside trees act as the frontline defense against urban stressors and are essential components of green infrastructure. They not only enhance biodiversity [8] and improve microclimate regulation by mitigating urban heat islands through their cooling effects [9], but also mitigate air pollution by absorbing heavy metals and particulate matter [2,7], consequently providing ecological, esthetic, and health benefits. However, prolonged exposure to urban stressors can impair tree vitality by affecting multiple functional traits, thereby hampering various cellular, biochemical, and physiological processes [10]. These include changes in morphological properties (e.g., leaf area, thickness, and stomatal density), phenological shifts (leaf initiation and development, flowering, fruiting, etc.), and disturbances in photosynthetic and water use efficiency, as well as changes in reactive oxygen species (ROS) metabolism, causing oxidative damage and disrupting the antioxidant defense system in plants [6,8,9]. Among these, photosynthesis is especially sensitive to urban stress. In particular, urban stress often leads to disruptions in carbon assimilation and sequestration, photochemical energy conversion, and stomatal regulation, resulting in reduced quantum yield and altered energy partitioning [8,11]. Stressed urban microclimates provoked by human activities and urbanization can induce oxidative stress in urban trees, thereby significantly impairing photosynthetic efficiency and affecting urban vegetation productivity [12,13,14].

However, the literature data evidenced that plants grown in urban settings employ various strategies to cope with environmental stressors [7,11]. Therefore, understanding how tree species respond to multiple, interacting stressors is essential for developing evidence-based strategies for urban greening and biodiversity conservation under future climate scenarios. Furthermore, modulations in plant functional traits can serve as critical bioindicators and biomarkers for assessing plants’ ability to withstand environmental stress, and can be used to assess plant susceptibility to emerging climate extremes [6,15,16,17]. For instance, monitoring chlorophyll fluorescence parameters enables the early detection of stress-induced limitations, providing valuable insights into the functional status and resilience of urban tree species, thus supporting the selection of resilient taxa for urban greening [18,19]. Although the physiological and biochemical responses of roadside trees have been widely studied, integrative approaches that capture a broad range of morphological, physiological, and biochemical indicators for assessing the tolerance of urban tree species to urban environmental stresses remain largely unexplored. A majority of recent studies have focused only on single stressors (e.g., isolated drought, air pollution, or heavy metal stress) rather than addressing the interactive effects of multiple co-occurring urban stressors, thereby limiting the comprehensiveness of urban tree tolerance assessments [6,20,21,22,23]. Therefore, such comprehensive evaluations are crucial for identifying resilient species suitable for urban greening, particularly in the face of projected climate change scenarios.

The primary objective of this study was to evaluate the physiological status of three ornamental tree species (*Tilia*, *Celtis*, and *Platanus*) growing along high-traffic urban roads and boulevards in Novi Sad. By comparing trees subjected to urban environmental stress with those from a minimally impacted reference site (control), we try to evaluate the plasticity of key leaf functional traits. This approach was designed to detect early physiological stress signals and capture seasonal variation in photosynthetic performance and antioxidant defense mechanisms. We hypothesized that structural, physiological, and biochemical responses of urban trees reflect their species-specific tolerance or sensitivity to multiple urban stressors. To address this, the study was guided by the following research questions: (i) How do chlorophyll fluorescence traits differ among tree species commonly used in urban greening? (ii) How does the timing of physiological and biochemical measurements (early vs. late summer) affect the detection of urban stress responses? (iii) Can specific physiological and biochemical indicators serve as reliable biomarkers for evaluating urban stress tolerance and support the development of predictive models for tree performance under urbanization?

## 2. Results

### 2.1. Structural and Water-Related Physiological Responses of Tree Species to Urban Stress

**Figure 1:**
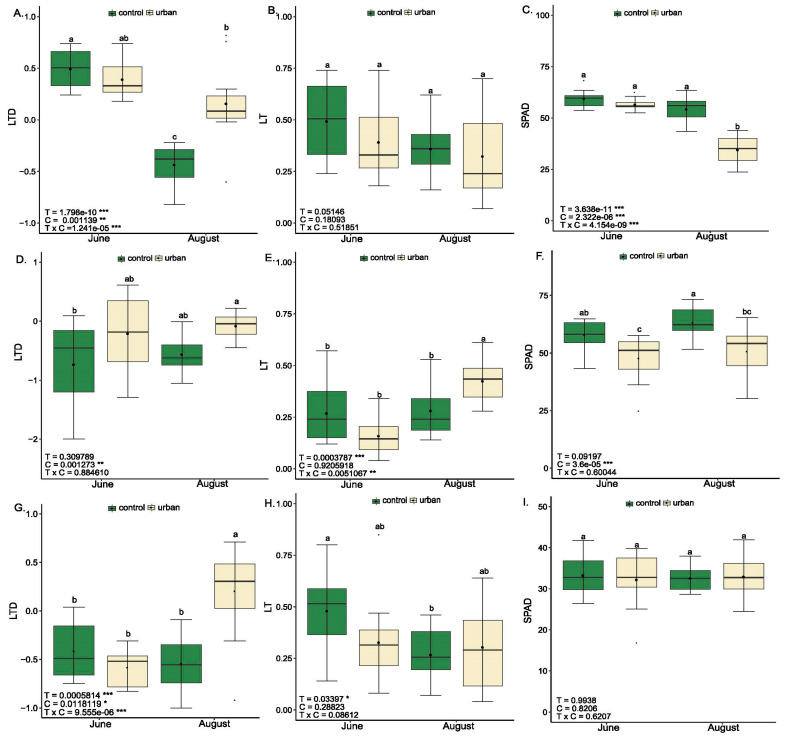
Structural and water status indicators of three urban tree species at two seasonal timepoints (June—baseline; August—stress peak) under control and urban conditions. Boxplots display measured parameters (LTD—Leaf Temperature Differential; LT—Leaf Thickness; SPAD—Relative Chlorophyll Content Index) for *Tilia* (**A**–**C**), *Celtis* (**D**–**F**), and *Platanus* (**G**–**I**). Data are presented as mean ± standard error (n = 12). Two-way ANOVA indicates the significance of the timepoint (T), condition (C), and their interaction (TxC). Different letters within the same species and trait indicate statistically significant differences according to Tukey’s HSD test (*p* < 0.05). Significance codes: * *p* < 0.05, ** *p* < 0.01, *** *p* < 0.001.

Analysis of differential leaf temperature, leaf thickness, and SPAD index (Relative Chlorophyll Content Index) revealed notable physiological and structural differences among the three studied urban tree species (*Tilia*, *Celtis*, and *Platanus*), influenced by environmental conditions (control vs. urban) and seasonal timing (June vs. August) (Figure 1).

Leaf Temperature Differential (LTD) significantly increased under urban stress conditions across all three species during the summer stress peak (Figure 1). In *Tilia*, urban trees exhibited a markedly higher LTD during the stress peak in August (*p* < 0.001), indicating impaired transpirational cooling. A similar pattern appeared in *Platanus* (Figure 1G), where LTD was elevated by 137.04% under urban conditions during the August stress period compared to the respective control, reaching its highest values (*p* < 0.001). In *Celtis* (Figure 1D), although the condition effect was significant (*p* < 0.01), urban effects were less pronounced, suggesting species-specific resilience to elevated temperatures.

Leaf thickness (LT) generally did not differ significantly between conditions, indicating relative anatomical stability across environments (Figure 1B,E,H). However, in *Celtis*, LT increased by 41.4% under urban conditions in August compared to the control, suggesting a seasonal structural adaptation. Additionally, seasonal effects on LT were significant in *Celtis* (*p* < 0.001) and *Platanus* (*p* < 0.05).

SPAD index values were significantly reduced under urban conditions in Celtis (*p* < 0.001), with decreases of 17.2% in June and 19.8% in August, indicating either chlorophyll degradation or inhibited chlorophyll biosynthesis. In *Tilia*, the lowest SPAD values were recorded in August under urban conditions, which were 34.4% lower than those of the control trees, highlighting pigment loss during summer peak stress. In contrast, *Platanus* did not show statistically significant changes in SPAD, emphasizing its physiological resilience to urban environmental pressures.

### 2.2. Photosynthetic and Fluorescence Responses of Tree Species to Urban Stress

Significant differences in photosynthetic efficiency and photochemical performance were observed across all three urban tree species in relation to environmental conditions (control vs. urban), seasonal timing (June vs. August), and their interaction (Table 1).

In *Tilia*, both condition and timepoint significantly affected *Φ*_II_ (Quantum Yield of Photosystem II), *Φ*_NO_ (Quantum Yield of Non-Regulated Energy Dissipation), *Φ*_NPQ_ (Quantum Yield of Regulated Non-Photochemical Quenching), *F*_v′_/*F*_m′_ (Ratio of Variable to Maximal Fluorescence (light-adapted), *g*_H_^+^ (Proton conductivity), and LEF (Linear Electron Flow) (*p* < 0.01), while *q*_L_ (Redox status of PSII) was influenced by condition (*p* = 0.02) and a strong interaction between condition and timepoint (TxC, *p* < 0.001). Notably, *Φ*_NO_ and *F*_v′_/*F*_m′_ displayed significant TxC interactions, highlighting complex seasonal dynamics across both control and urban environments (Table 1).

*Celtis* showed fewer main effects from condition or timepoint alone; however, significant TxC interactions were found for *Φ*_II_ (*p* = 0.0032), *Φ*_NO_ (*p* = 0.0012), and *q*_L_ (*p* = 0.00078), indicating a combined impact of urban stresses related to seasonal dynamics. LEF was particularly sensitive to urban stress, showing a highly significant treatment effect (*p* = 8.6 × 10^−11^) and a 62.33% decrease during peak summer stress under urban conditions.

In *Platanus*, significant seasonal effects (June vs. August) were detected for most parameters, including *Φ*_II_, *Φ*_NO_, *Φ*_NPQ_, *F*_v′_/*F*_m′_, and *g*_H_^+^ (*p* < 0.01). All of these parameters, except *Φ*_NPQ_, also exhibited significant condition effects (Table 1). LEF was influenced by both condition (*p* = 0.02) and TxC interaction (*p* = 0.031). Conversely, v_H_^+^ (Proton flux) and *q*_L_ remained stable, showing no significant effects from condition, season, or their interaction.

### 2.3. Oxidative Stress Markers and Antioxidative Enzyme Activities of Tree Species to Urban Stress

Significant alterations in oxidative stress biomarkers and antioxidant enzyme activities were observed across the analyzed tree species in response to urban environmental conditions, particularly during the peak summer stress period (Table 2). Lipid peroxidation, measured as TBARS, exhibited a marked increase under stress conditions, particularly during the summer peak stress period (*p* < 0.001 for all tested plant species). In *Tilia*, TBARS levels increased more than sixfold from June, under control conditions (4.02 nmol TBARS/mg protein), to the urban stress peak (27.07 nmol TBARS/mg protein). *Platanus* exhibited a similar pattern, showing a significant increase by 71.7% in TBARS in urban conditions during August, confirming elevated oxidative stress in urban conditions. However, *Celtis* exhibited a moderate but significant increase of 37.2% in TBARS level during August (Table 2). The same trend was observed in proline content, which increased significantly under urban conditions regardless of seasonal timing in *Tilia* and *Platanus*, indicating active stress mitigation (Table 2). The highest increase in proline levels was recorded during August in *Tilia* under urban conditions, with a 5.62-fold rise compared to the control. Meanwhile, *Celtis* exhibited notable seasonal variation (*p* < 0.001) but no clear condition effect, suggesting a different regulatory mechanism. Glutathione, another key redox regulator, also increased significantly in *Tilia* and *Celtis* under urban conditions during August (*p* < 0.001), pointing to an enhanced antioxidative response. *Platanus* showed seasonal variation (*p* < 0.01) but no significant condition effect (Table 2).

APX activity increased significantly in all species under urban conditions (*p* < 0.001), except for *Platanus* during June, where APX activity was maintained at the same level as in the control (Table 2). CAT activity was also significantly higher under urban stress in *Tilia* (a 2-fold increase) and *Celtis* (a 4-fold increase) during August. In contrast, *Platanus* showed a decrease in CAT activity under urban conditions; however, according to Tukey’s post hoc test, the changes during specific seasonal periods were not statistically significant (Table 2).

**Figure 2:**
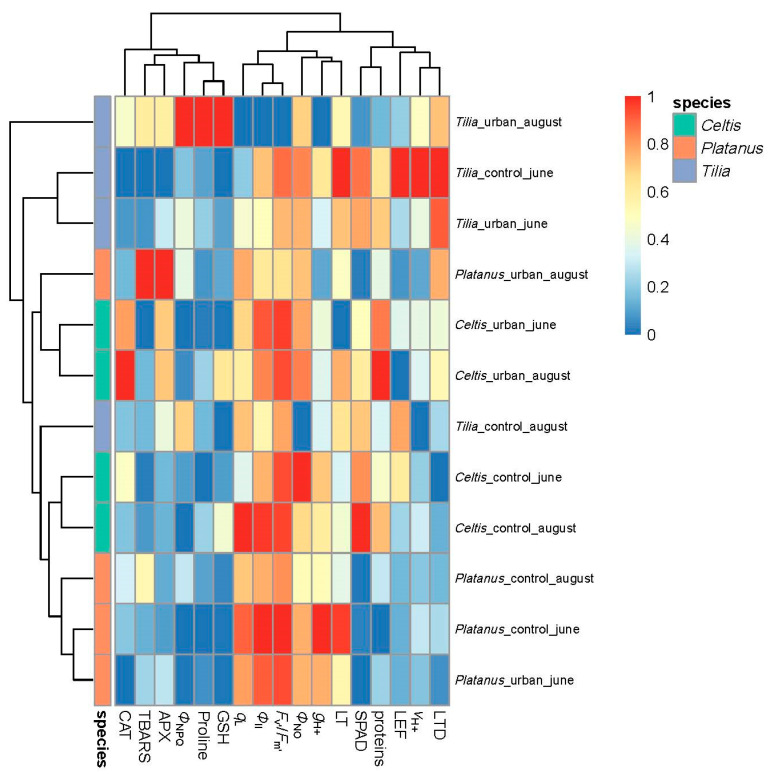
Clustered heatmap of normalized structural, physiological, and biochemical traits across *Tilia*, *Celtis*, and *Platanus* under different conditions (urban vs. control) and timepoints (June vs. August). Traits were scaled using min–max normalization, and hierarchical clustering was performed on both traits and treatment combinations. Color annotations indicate tree species.

The heatmap revealed distinct species-specific clustering of structural, physiological, and biochemical traits, revealing the plant’s functional profile under urban stress conditions (Figure 2). *Tilia* showed a most distinct separation between urban and control conditions, particularly during August, indicating high sensitivity to urban stress. On the contrary, *Platanus* showed more consistent trait responses between control and urban conditions, forming a more compact cluster, with slight alterations in the quantum yield of PSII and antioxidative response under urban conditions in August. Meanwhile, *Celtis* displayed intermediate clustering, where urban conditions separate from control due to a shift in antioxidative enzyme activity and chlorophyll fluorescence traits (Figure 2).

At the trait level, stress-indicative biomarkers such as TBARS, APX, CAT, and proline contributed strongly to the separation of urban-exposed plants from conditions, particularly in *Tilia* and *Celtis*. Traits associated with photosynthetic performance (*Φ*_II_, *F*_v′_/*F*_m′_, LEF, *q*_L_) and leaf water dynamics (LT, LTD) were generally decreased in urban conditions, especially in *Tilia*, supporting the species’ higher sensitivity to urban-induced physiological stress. Overall, clustering by species and condition highlighted divergent stress-coping strategies, with *Platanus* exhibiting the most robust profile under urban stress.

### 2.4. Tree Health Risk Index of Tree Species to Urban Stress

**Figure 3:**
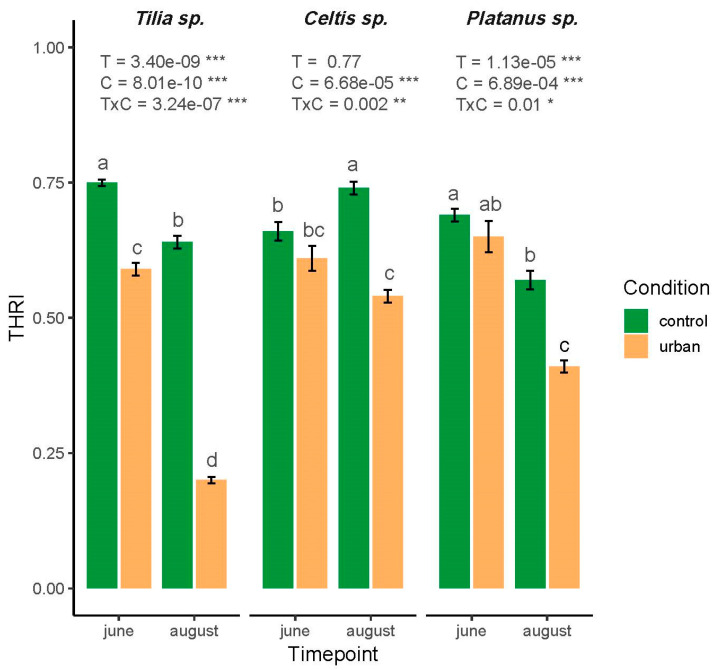
Tree Health Risk Index (THRI) of three urban tree species (*Tilia*, *Celtis*, and *Platanus*) under control and urban conditions across two seasonal timepoints (June and August). Values represent mean ± SE (*n* = 3). Significant effects were observed for condition (C), timepoint (T), and their interaction (TxC) as indicated by two-way ANOVA results. Letters indicate statistically significant differences among treatments based on Tukey’s HSD post hoc test (*p* < 0.05). Significance codes: * *p* < 0.05, ** *p* < 0.01, *** *p* < 0.001.

The Tree Health Risk Index, calculated by integrating structural, physiological, and biochemical traits, revealed species-specific responses to urban environmental stress and seasonal variation (Figure 3). THRI in *Tilia* showed a significant main effect of timepoint, condition, and their interaction (*p* < 0.001). THRI was the highest in June under control conditions (0.75) and declined markedly in urban conditions during August (0.2), indicating high sensitivity to the co-occurring stresses during the summer period. In contrast, *Celtis* exhibited a significant effect of condition (*p* < 0.001) and interaction (*p* = 0.002), but not of timepoint (Figure 3). Moderate reductions in THRI were observed under urban conditions, although the changes were not significant in June, whereas a significant decline was recorded in August. *Platanus* displayed a similar pattern to *Tilia*, with a significant effect of timepoint (*p* < 0.001), condition (*p* < 0.001), and their interaction (*p* = 0.012), although the changes were less pronounced. The highest THRI value was recorded in June under control conditions, with notable declines in August under urban settings. Moreover, post hoc Tukey tests confirmed significant pairwise differences among condition–timepoint combinations for each species (*p* < 0.05), with *Tilia* showing the most pronounced shifts, followed by *Celtis* and *Platanus* (Figure 3).

**Figure 4:**
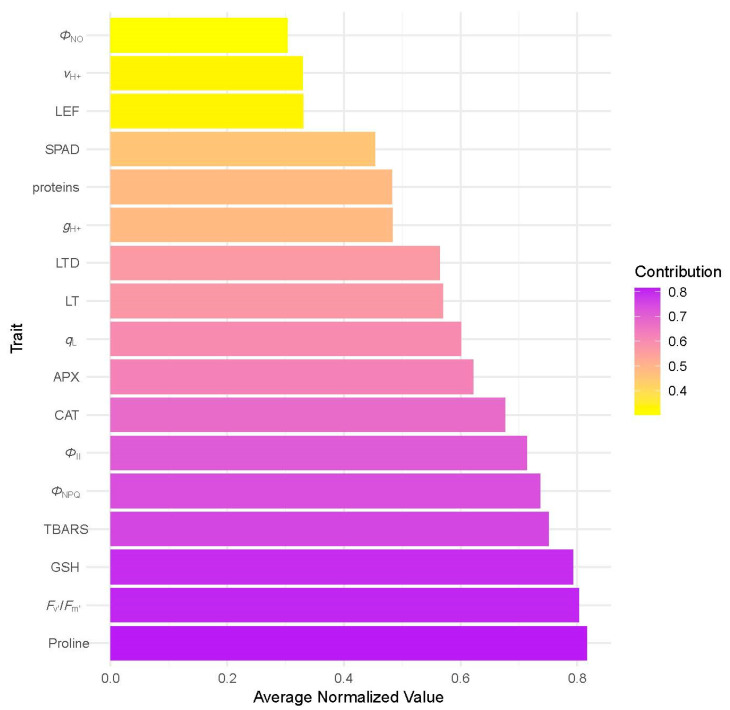
Relative contribution of individual structural, physiological, and biochemical traits to overall THRI values, based on average normalized scores.

The relative contribution of individual structural, physiological, and biochemical traits to overall THRI values is presented in Figure 4, showing that Proline, *F*_v′_/*F*_m′_, and GSH had the highest normalized mean values, reflecting a substantial impact on the overall THRI scores and emphasizing their importance in resilience assessment. Conversely, parameters such as *Φ*_NO_, *v*_H_^+^, and LEF contributed less to the overall tree health risk index. These findings suggest that both photosynthetic performance and oxidative stress markers play significant roles in shaping tree health profiles under urban stress.

## 3. Discussion

Urbanization has a notable impact on urban vegetation productivity by affecting photosynthesis and greenness, which are driven by multiple climatic and environmental factors. The foliage of roadside trees is continuously exposed to air pollution, vehicular emissions, elevated temperatures, and episodic summer droughts, all of which can severely disrupt photosynthetic efficiency and compromise plant vitality and ecosystem functioning [18,22,23,24]. Given their continuous exposure to multiple co-occurring stressors, urban trees provide a relevant model for assessing photosynthetic responses to complex environmental conditions. In the face of environmental stresses, altered environments can mirror projected future climate scenarios, including higher air temperatures, increased atmospheric CO_2_ levels, and elevated levels of air pollutants [3,15]. On the other hand, the complexity of plant–environment interactions in urban ecosystems, where numerous abiotic, biotic, and anthropogenic disturbance factors act simultaneously, poses a challenge in identifying consistent trends in plants’ functional trait responses [19]. Detectable trait shifts typically emerge only when a dominant stressor elicits a strong response or when multiple stressors exert synergistic effects in the same direction [25].

This study demonstrates the effects of seasonal variation in growing conditions on structural, physiological, and biochemical leaf traits in urban tree species, highlighting both species-specific sensitivity and adaptive plasticity, especially during high-stress summer periods. The elevated LTD values under urban conditions reflect a compromised ability of leaves to regulate leaf temperature, most likely due to impaired stomatal conductance or reduced water availability. This was especially notable in *Tilia* and *Platanus*, where higher LTD during the stress peak aligns with intensified urban heat and water limitation. These findings confirm previous observations that leaf temperature serves as a sensitive early indicator of heat and water stress. Consistent with our findings, You et al. (2016) [26] reported elevated leaf temperatures in two roadside species, *Platanus occidentalis* and *Ginkgo biloba*, growing at a polluted site, suggesting reduced transpiration rates compared to trees at a less polluted site. Recently, Mitchell et al. (2025) [17] confirmed that leaf thermoregulation is facilitated by effectively controlled transpiration, which maintains leaf temperature at near-optimal levels for photosynthesis. Additionally, Ilyas et al. (2021) [2] reported that the plasticity of leaf functional traits, particularly those related to stomatal structure and functioning, can reflect urban habitat quality and thus be used as early stress indicators.

Increased leaf thickness in stressed urban trees likely represents a structural acclimation strategy, possibly via thicker mesophyll layers providing efficient light capturing and carbon assimilation or by enhanced cuticular development to mitigate water loss [27]. In our study, urban stress during August induced an elevation in leaf thickness in *Celtis* compared to the control (Figure 1), suggesting that thicker leaves are associated with enhanced drought tolerance and a greater internal water storage capacity, which was further associated with relatively stable PSII efficiency. Our results support previous findings that leaf thickness is a crucial functional leaf trait influencing the fitness of species, indicating that plants employ different strategies to cope with adverse environmental conditions [27,28]. Moreover, Kisvarga et al. (2023) [8] reported that inherent defense mechanisms against abiotic stress involve shifts in morphological traits, such as increased leaf thickness and decreased stomatal density, followed by physiological adjustments, including restoring osmotic balance, closing stomata, and synthesizing antioxidant molecules and enzymes.

SPAD index patterns suggest a decline in photosynthetic pigment content under urban stress, particularly in *Tilia* during August, and it was also evident in *Celtis*, regardless of seasonal timing (Figure 1). Such reductions can indicate oxidative damage or downregulation of chlorophyll synthesis, both of which are typical under combined heat and drought stress. Conversely, *Platanus* maintained SPAD index level, suggesting greater pigment stability, which could be indicative of more efficient antioxidant defenses or photoprotective strategies. Consistent with our findings, Andrianjara et al. (2024) [10] reported a typical chlorotic phenotype in *Tilia cordata* exposed to trace element pollution and soil water deficit, supporting the use of chlorophyll content as a reliable indicator of multiple stress conditions. However, inconsistent with our findings, Yan et al. (2025) [29] observed an elevation in the SPAD index across commonly grown urban trees (*Acer pictum*, *Fraxinus chinensis*, *Koelreuteria paniculata*, *Salix babylonica*, *Sophora japonica*) along an urban–rural gradient. These inconsistencies among studies may be attributable to interspecies differences, as well as to the consistency and intensity of urban stressors [25,29].

Photosynthesis is among the most sensitive physiological processes in plants, particularly vulnerable to cumulative stress factors in urban environments, such as heat, drought, and pollution [15,19]. Moreover, our previous research has highlighted the importance of microhabitat conditions in shaping plants’ responses and acclimatization to multiple environmental pressures, primarily reflected in variations in photosynthetic intensity and photosystem II efficiency [16]. In this study, apparent interspecific differences were observed in the photochemical and physiological responses of three commonly planted urban tree species to seasonal and environmental changes. These differences underscore species-specific strategies and sensitivities to urban stress.

In *Tilia*, significant reductions in *Φ*_II_ and LEF under urban conditions, especially in August, indicate impaired photochemical efficiency and electron transport, which can be attributed to the unfavorably dry weather conditions during the summer period. A simultaneous increase in *Φ*_NPQ_ suggests activation of photoprotective thermal dissipation mechanisms to mitigate damage from excess light energy. A strong negative correlation between the quantum yield of photosystem II and non-photochemical quenching is often observed under various environmental stress conditions, such as excess light, drought, elevated temperatures, and salinity, reflecting the plant’s photoprotective response to impaired photosynthetic efficiency [30]. The significant interaction effects for *F*_v′_/*F*_m′_, LEF, and *Φ*_NO_ (TxC; *p* < 0.05) highlight that both urban conditions and seasonal timing synergistically exacerbate stress, indicating *Tilia*’s higher sensitivity and dynamic photochemical response. Consistent with our findings, Kościesza et al. (2025) [24] reported that *Tilia × europaea* ‘Pallida’, when grown along high-traffic roadsides, exhibited lower resistance to urban stressors, such as air pollution, elevated summer temperatures, and wind exposure, compared to *Alnus cordata*, as reflected by reduced photosynthetic efficiency and a shorter foliage lifespan under variable weather conditions. Meanwhile, *Celtis* demonstrated moderate responses. While *Φ*_II_ remained relatively stable across treatments, indicating a potential buffering capacity or delayed onset of stress symptoms, significant changes in *Φ*_NO_, LEF, and *q*_L_, particularly under urban conditions in August, suggest adjustments in energy partitioning and redox status. Notably, LEF declined by more than 60% under urban stress, which may indicate impaired carbon fixation capacity. These findings suggest that *Celtis* possesses an intermediate resilience strategy that partially sustains photochemical integrity under prolonged urban exposure. Our results align closely with data from Popek et al. (2018) [12], who reported a significant adverse effect on gas exchange parameters, including CO_2_ assimilation and stomatal conductance, while the maximum quantum efficiency of photosystem II remained unchanged in roadside woody plants growing in urban environments. Moreover, *Celtis australis* was previously recognized as a species with an effective isohydric strategy characterized by physiological and biochemical adjustments. These include efficient down-regulation, rather than permanent impairment of PSII photochemistry, along with increased activity of antioxidant enzymes, to prevent irreversible damage to the photosynthetic apparatus under drought stress [11]. In contrast, *Platanus* exhibited a pronounced decline in *F*_v′_/*F*_m′_ and *g*_H_^+^ during August under urban stress, indicating compromised PSII efficiency and ATP synthase activity. Nevertheless, the stability of *v*_H_^+^ and *q*_L_ across treatments suggests a degree of buffering in proton flux and redox equilibrium. The lack of variation in these parameters, despite broader stress signals, may reflect a protective strategy in which *Platanus* maintains core photoprotective and redox-regulatory functions to preserve its baseline photosynthetic activity. Stable SPAD values accompany the observed changes, suggesting that photochemical efficiency and ATP synthase activity were impaired under stress conditions without corresponding chlorophyll degradation. This indicates a functional limitation in PSII and thylakoid energy conversion, rather than a structural loss of photosynthetic pigments, potentially reflecting an early or adaptive response to environmental stress before pigment content is affected [31]. Moreover, the results suggest that chlorophyll fluorescence parameters serve as reliable functional traits for the early detection of abiotic stress, due to their high sensitivity to environmental changes.

Taken together, these results emphasize that while *Tilia* exhibits high plasticity but reduced tolerance under urban stress, *Celtis* displays moderate resilience, and *Platanus* employs a conservative strategy by preserving key regulatory pathways. Traits such as *Φ*_NPQ_, LEF, and *Φ*_NO_ emerged as sensitive and responsive indicators of urban-induced photoinhibition and may serve as reliable markers for early stress detection. In contrast, parameters like *V*_H_^+^ and *q*_L_ may reflect longer-term stability rather than immediate stress responsiveness.

It is well known that the climate change resilience of plants is closely linked to leaves’ ability to endure environmental stresses, as leaves are the primary site of photosynthesis [17]. It is well established that the components of PSII, particularly the D1 protein and associated reaction center complexes, are highly sensitive to heat stress. Elevated temperatures can disrupt the structural integrity and functional efficiency of PSII, thereby impairing photochemical energy conversion and leading to reduced photosynthetic performance. This thermosensitivity makes PSII a reliable indicator of heat-induced physiological stress in plants, especially under urban and climate-related stress conditions [17]. However, the impact of environmental stressors on plant functional traits can vary in consistency and predictability, depending on the specific nature of the stressor and the extent of its influence on plant physiological and structural responses [25]. The findings of this study support the above-mentioned points, underscoring the importance of species-specific selection in urban forestry and the utility of chlorophyll fluorescence traits as non-invasive diagnostic tools for assessing plant health and photosynthetic resilience under changing environmental conditions.

The biochemical responses observed across *Tilia*, *Celtis*, and *Platanus* reflect complex and species-specific stress adaptation strategies under urban environmental conditions. The results highlight species-specific oxidative stress responses, with *Tilia* showing the most pronounced biochemical sensitivity and activation of enzymatic defenses, implying lower resilience but stronger compensatory mechanisms. *Celtis* and *Platanus* exhibited varying degrees of enzymatic and non-enzymatic responses, indicating distinct adaptive strategies to urban-induced oxidative stress. The consistent elevation of TBARS across species (Table 2) indicates intensified lipid peroxidation, likely due to more pronounced urban stressors during summer peaks. This aligns with previously reported findings that reveal trends in oxidative damage occurring in plants exposed to urban conditions [6,9]. The rapid and substantial production of reactive oxygen species (ROS) is one of the earliest and most prominent responses plants exhibit when exposed to stress conditions. Stress-induced ROS are highly reactive and can cause multiple oxidative damages to cellular components, including lipids, proteins, and nucleic acids, as well as cell membranes, which can be evidenced by TBARS production [32]. However, plants have developed a plethora of integrated biochemical adjustments to effectively combat abiotic stressors, including osmotic adjustment and the production of low-molecular-weight molecules, as well as the activation of enzymatic and non-enzymatic antioxidants [10,11]. The overproduction of compatible osmolytes, such as proline, is considered an effective strategy for mitigating oxidative stress. Proline is a versatile stress metabolite that contributes to ROS detoxification by scavenging free radicals, stabilizing cell membranes, and maintaining the structure and function of proteins [33]. In this study, we recorded a pronounced increase in proline content in *Tilia* under urban conditions during August (Table 2), highlighting its vital role in osmoprotection and ROS scavenging mechanisms in response to environmental stress. Notably, *Celtis* and *Platanus* also demonstrated enhanced proline biosynthesis during August, although to a lesser degree. Our results align with those of Andrianjara et al. (2024) [10], indicating that proline and malondialdehyde (MDA), an end-product of lipid peroxidation, may serve as sensitive early indicators of biochemical stress responses in *Tilia* species under water-deficit conditions. These markers can reveal stress well before visible leaf symptoms occur, offering valuable tools for urban green space managers to monitor tree health. Moreover, plants possess a complex network of enzymatic (e.g., superoxide dismutase, polyphenol oxidase, peroxidase, ascorbate peroxidase, catalase, and glutathione reductase) and non-enzymatic antioxidants (β-carotene, ascorbate, α-tocopherol, glutathione, and carotenoid) that act together to neutralize ROS and diminish oxidative stress in plants. The most pronounced APX and CAT upregulation observed in *Tilia* and *Celtis* suggests a more active H_2_O_2_ detoxification system in these species, possibly reflecting differences in peroxisome dynamics or substrate availability. GSH accumulation further complements this defense strategy, particularly in *Tilia*, supporting the glutathione–ascorbate cycle as a critical response under oxidative stress. Overall, the obtained data underscore the necessity of species-specific physiological profiling of urban trees and highlight the importance of using integrative biochemical indicators to monitor and manage the vitality of urban trees and their tolerance to withstand environmental stress.

Based on the observed clustering patterns, it can be highlighted that species-specific resilience and adaptive capacity to urban stressors rely on versatile structural, physiological, and biochemical modifications. Among the three studied species, *Tilia* exhibited the most distinct separation between urban and control conditions, particularly in August. This divergence suggests heightened sensitivity to prolonged summer stress, consistent with earlier findings indicating that *Tilia* species often show pronounced reductions in photosynthetic efficiency and antioxidant capacity in polluted or heat-stressed environments. The clustering of *Tilia* urban samples with elevated levels of TBARS, proline, and antioxidant enzymes (APX, CAT) supports this conclusion, as these biomarkers are commonly associated with oxidative stress and cellular damage. *Celtis* displayed an intermediate response, with clustering patterns suggesting moderate plasticity in trait expression under urban influence. While stress markers such as TBARS and proline increased under urban conditions, the magnitude of change was less pronounced than in *Tilia*. This species’ moderate clustering suggests that while *Celtis* does experience stress, its coping mechanisms may be more adaptive than those of *Tilia*, though less robust than those of *Platanus*. In contrast, *Platanus* exhibited a less pronounced separation between control and urban settings, indicating a relatively stable physiological profile across different environments. This may reflect a more effective or constitutive defense strategy, enabling better maintenance of photosynthetic performance and oxidative balance under stress. Previous studies have noted the robust morphological plasticity and physiological tolerance of *Platanus* in urban ecosystems, which aligns with the patterns observed in this study [5,9]. For instance, *Platanus x hispanica* is a fast-growing species, highly resistant to urban microclimate conditions such as soil compaction and air pollution, possessing a high phenotypic plasticity, resistance to frost, drought, and moderate wind regimes, making it a good candidate for urban forests in cities worldwide with different climatic types [5].

The trait-level clustering further revealed that oxidative stress markers (TBARS, APX, CAT, proline) consistently contributed to urban–control differentiation, reinforcing their utility as reliable indicators of environmental stress. In contrast, traits such as *Φ*_II_, *F*_v′_/*F*_m′_, and *q*_L_ were reduced in urban samples, confirming the detrimental impact of urban environments on photosynthetic efficiency.

The integrative THRI approach provided a robust, composite assessment of tree vitality, capturing functional traits differences across tree species and urban contexts. Also, such indicators are essential in ecophysiological research, as they help disentangle complex interactions between trees and multiple stress-inducing factors, while improving the assessment of overall tree and ecosystem health [34]. In our study, the notable decline in THRI observed in August under urban conditions likely reflects the cumulative effects of low water availability, intensified heat, and pollution stress in urban microclimates during peak summer. These factors induced elevation of oxidative stress and reduced photosynthetic efficiency, resulting in lower integrative vitality scores. According to the study by Callow et al. (2018) [35], a strong positive correlation was found between leaf water potential and the urban visual vitality index, as well as between leaf water potential and bark chlorophyll fluorescence, in Elm trees (*Ulmus* spp.) grown in urban environments exposed to drought stress. Likewise, Sepúlveda and Johnstone (2019) [36] observed statistical relationships between bark and leaf chlorophyll fluorescence and pre-dawn water potential in *Ficus macrophylla* and *Platanus × acerifolia*, while no correlation was observed in *Ulmus parvifolia*. Simultaneously, the authors observed almost no statistically significant relationship between leaf and bark chlorophyll fluorescence and the visual vitality index in mature *Platanus × acerifolia* trees. These findings highlight the species-specific sensitivity of this indicator, underscoring the need for integrative indices that incorporate a broader range of physiological and biochemical parameters to offer a more comprehensive and reliable evaluation of long-term stress impacts on urban trees. To the best of our knowledge, our study is the first to report the resilience of *Tilia*, *Celtis*, and *Platanus* in an urban stressed environment, based on THRI assessment, which highlights the synergistic effects of urban environmental stressors on photosynthetic regulation and antioxidant responses. However, the existing literature includes various composite indices that reflect tree health in urban green areas, primarily based on crown and vegetation indices, which focus on chlorophyll fluorescence traits [37,38]. Similar to our study, Singh (2023) [6] reported an integrated approach that considers physiological and biophysical-based indicators for assessing the tolerance of *Alstonia scholaris* roadside plantations to urban roadside air pollution. In our study, *Tilia* demonstrated the most significant seasonal and environmental sensitivity, confirming its vulnerability to summer urban stressors. This aligns with previous observations of declined leaf gas exchange and antioxidant imbalances in *Tilia* under drought and pollution [10,24]. *Celtis*, while generally more resilient, exhibited lower overall THRI under urban conditions, suggesting a latent stress accumulation or altered acclimation potential. *Platanus* exhibited intermediate behavior, likely due to its known adaptability to urban climates, but with clear limits under compounded summer stress. The trait-wise contribution to THRI (Figure 4) highlighted that Proline, *F*_v′_/*F*_m′_, and GSH were the most influential parameters, suggesting their strong potential in reflecting tree vitality. Conversely, traits such as *Φ*_NO_, *v*_H_^+^, and LEF had relatively lower contributions, although they may still hold importance in specific physiological contexts or species. Interestingly, the high levels of Proline and TBARS, both known biochemical markers of oxidative stress, indicate that stress-related biochemical responses play an important role in determining the composite health status of urban trees. This is consistent with the observed decline in THRI in August, when oxidative stress is likely to be more pronounced due to elevated temperatures and drought conditions. Similarly, the strong contribution of F_v′_/*F*_m′_, a chlorophyll fluorescence parameter reflecting PSII efficiency, underscores the sensitivity of the photosynthetic apparatus to environmental perturbations and its suitability for early stress detection. Taking all into account, THRI proved to be an effective tool for quantifying integrative plant stress responses. Although THRI requires the use of expensive devices that are mainly limited to well-equipped and funded laboratories, the adoption of new technologies, including low-cost devices such as MultispeQ [39], demonstrates its potential for simplification and adaptation for routine urban tree assessments. Therefore, THRI can serve as a valuable conceptual framework for identifying key physiological indicators related to urban stress and guiding management decisions, such as irrigation optimization or species selection for improved resilience and urban ecosystem service. Additionally, future integration with remote sensing or real-time sensor platforms may enhance its application for precision arboriculture and sustainable urban greening strategies. In addition, including soil physicochemical properties such as nutrient availability, pH, and moisture in future assessments could further enhance the sensitivity and accuracy of the THRI in reflecting the overall health status of urban trees.

Although our study distinguished *Platanus* as the most resilient species to urban environmental stressors, making it a strong candidate for long-term urban greening and stress mitigation strategies, additional factors should also be considered in urban planning. These include its susceptibility to pests and diseases, as well as the emission of volatile organic compounds and allergens, which are particularly relevant in the context of climate change challenges that cities are facing [5].

## 4. Materials and Methods

### 4.1. Study Sites and Plant Material

The study was conducted in Novi Sad, Serbia (45°15′15″ N, 19°50′33″ E), at three urban localities characterized by tree-lined avenues: Futoška Street, with *Platanus × hispanica* Mill. ex Münchh. (London plane); Radnička Street, with *Celtis occidentalis* L. (Common hackberry); and Bulevar Jovana Dučića, with *Tilia platyphyllos* Scop. (Largeleaf linden). These locations were selected based on their aged tree lines and high exposure to urban stressors (heavy vehicular traffic, air pollution, noise, prolonged drought, and heat waves during the summer period). The Danube Park (Novi Sad, Serbia) was selected as a control site since it represents a monument of nature and a protected natural resort. Therefore, it is protected from direct traffic exposure, representing a low-stress environment. The same tree species investigated at the roadside locations are present in this park under comparable environmental conditions, except for the absence of vehicular emissions. Additionally, the trees in the park are regularly irrigated, particularly during the summer months, to maintain optimal soil moisture levels and minimize drought stress. At each location, five mature trees of each species were randomly selected for analysis, ensuring comparable age, canopy development, morphological uniformity, and health status. Tree selection was based on a visual assessment of overall vitality, ensuring that only individuals without pest damage, disease symptoms, or recent pruning scars were included. Based on diameter at breast height (DBH) measurements and local planting records, the selected trees were mature specimens representing typical roadside plantings: *Celtis occidentalis* (DBH = 49.6 cm; ~100 years), *Tilia platyphyllos* (DBH = 30 cm; ~40 years), and *Platanus × hispanica* (DBH = 55.7 cm; ~50 years). Physiological and biochemical parameters were measured on fully expanded, sun-exposed leaves.

Plant sampling was conducted at two critical points in the vegetation season: (i) early June 2024, representing the physiological optimum, with fully developed canopies, optimal water availability, and an average air temperature of 24.3 °C, accompanied by 57.3 mm of rainfall. (ii) August 2024, capturing summer peak environmental stress during a prolonged dry period, with air temperatures averaging 27.5 °C and an extremely arid period with only 2.2 mm of rainfall. According to data from the Serbian Hydrometeorological Service, August 2024 was recorded as the hottest August in Serbia since 1951. In Novi Sad, the maximum daily air temperature reached 40.2 °C, marking a new historical record for the city (www.hidmet.gov.rs, accessed on 15 July 2025) [40].

### 4.2. Photosynthesis-Related Parameters

The functional status of the photosynthetic apparatus and associated stress indicators were evaluated using the MultispeQ V 2.0 device (PhotosynQ Inc., East Lansing, MI, USA), following the standardized Photosynthesis RIDES 2.0 protocol. Measurements were conducted on intact, fully developed leaves, with twelve replicates per treatment. Both fluorescence-based and absorbance-based parameters were recorded to assess the efficiency of PSII, photoprotective mechanisms, energy dissipation, and water status. All measurements were conducted on fully expanded, sun-exposed leaves on clear, sunny days between 9:00 and 12:00 to ensure stable light conditions and minimize diurnal variation. Measurements were first performed in urban sites, followed by the control (park) site, under comparable irradiance and temperature conditions (with differences not exceeding 45 min between sites). This approach minimized potential effects of midday photoinhibition and ensured data comparability across species and locations. The complete list of measured parameters and their physiological relevance is provided in Table 3. The LTD was calculated as the difference between leaf and ambient temperature, serving as an indicator of transpirational cooling efficiency. The linear electron flow (LEF) was calculated according to Kuhlgert et al. (2016) [39] using the following equation:

LEF = *Φ*_II_ × PAR × 0.45 (1)

0.45 is a factor that approximately accounts for the absorptance of PAR and the fraction of absorbed light that is transferred to PSII centres.

### 4.3. Biochemical Stress Markers and Enzymatic Antioxidant Response

To evaluate enzymatic, non-enzymatic antioxidants, and biochemical stress markers, fresh leaves (0.2 g) were placed in liquid nitrogen and stored at −80 °C prior to analysis. The frozen leaves were homogenized with 2 mL of 50 mmol/L potassium phosphate buffer (pH 7.0), containing 1 mmol EDTA and 1% polyvinylpyrrolidone (PVP). The homogenate was centrifuged at 12,000× *g* for 10 min at 4 °C, and the supernatant was used for enzymatic assay measurements. Additionally, the supernatant was used to quantify soluble proteins using Bradford’s method (1976) [46], with bovine serum albumin as a standard.

Ascorbate peroxidase (APx, E.C. 1.11.1.11) activity was determined spectrophotometrically by monitoring the reduction in absorbance at 290 nm following the addition of H_2_O_2_, according to Nakano and Asada (1981) and Amako et al. (1994) [47,48]. Catalase (CAT, EC 1.11.1.6) activity was measured by monitoring the decomposition of H_2_O_2_ at 240 nm [49]. The results are expressed in units (U) per milligram of protein.

The content of reduced glutathione (GSH) was quantified using the method described by Kapetanović and Mieyal (1979) [50]. Plant samples (0.5 g) were homogenized in a 5% sulfosalicylic acid solution and centrifuged at 3000 rpm for 10 min. The supernatant was mixed with Ellman’s reagent, and the absorbance was measured at 412 nm after a 5 min reaction period. GSH concentration was reported as µmol of reduced glutathione per mg of protein. Proline content was determined according to Bates et al. (1973) [51], with adaptations from Lee et al. (2018) [52], using sample aliquots extracted from 5% sulfosalicylic acid. The supernatant was mixed with a 1.25% ninhydrin solution in 80% acetic acid. The mixtures were incubated at 100 °C for 30 min, and the absorbance was measured at 595 nm. Proline content was expressed as nmol per gram of fresh plant weight. Lipid peroxidation was assessed by quantifying thiobarbituric acid reactive substances (TBARS) according to Devasagayam et al. (2003) [53]. A 0.25 mL plant extract was mixed with 2.25 mL of TBA (thiobarbituric acid), 10% PCA (perchloric acid), and 20% TCA (trichloracetic acid) solution, incubated at 95 °C for 30 min, then centrifuged (3000 rpm, 10 min). The absorbance of the supernatant was measured at 532 nm, and the results were expressed as nmol TBARS per mg protein.

Each biochemical parameter was measured in triplicate. The absorbance of each parameter was assessed in 96-well plates using a spectrophotometer (Multiskan GO, Thermo Fisher Scientific, Waltham, MA, USA).

### 4.4. Tree Health Risk Index (THRI) Calculation

To evaluate the overall health status of urban trees under different environmental conditions, we calculate a Tree Health Risk Index (THRI) by integrating structural, physiological, and biochemical traits. The THRI provides a synthetic indicator of urban tree vitality, enabling early detection of stress-induced decline and supporting species selection and management strategies in urban environments. The parameters used in this calculation included SPAD, *Φ*_II_, *Φ*_NO_, *Φ*_NPQ_, *F*_v′_/*F*_m′_, *g*_H_^+^, *v*_H_^+^, LEF, *q*_L_, LTD, LT, TBARS, Proline, GSH, total proteins, APX, and CAT. All traits were first normalized using Min-Max scaling to a range between 0 and 1. For traits where higher values indicate greater stress (TBARS, Proline, *Φ*_NO_, *Φ*_NPQ_, LTD, GSH, APX, and CAT), the values were inverted (1-x) after normalization to ensure that higher normalized values represent better plant health. This approach ensures an integrative score that reflects the cumulative physiological status of the trees and was adapted from similar ecological health and risk indices [6].

The THRI calculation is based on a normalized trait matrix:

$$THRI=\frac{1}{n}\sum_{j=1}^{n}Zij$$

THRI—Tree Health Risk Index for the i-th condition/species/timepoint group

Zij—Normalized value of the j-th trait for the i-th group

n—Total number of traits included in the index

To determine the trait-specific contribution to THRI, we computed the mean normalized value for each trait across all samples. These values were visualized using barplots to assess which traits most strongly influenced the composite health index.

### 4.5. Data Analysis

All data generated and analyzed are presented in Table S1. All statistical analyses and visualization were performed using RStudio version 4.2.3 (R Core Team, 2023) [54], and results are presented as mean ± standard error (SE). Bartlett’s test and the Shapiro–Wilk test were used to assess the homogeneity of variance and the normality of data, respectively. To evaluate the effects of condition (urban vs. control), timepoint (June vs. August), and their interaction, a Two-way ANOVA was performed for each species. When significant effects were detected, Tukey’s HSD or Sidak-adjusted post hoc tests were applied, using car, emmeans, and multicompView packages. Detailed ANOVA output tables for each parameter are provided in the Supplementary Information (Tables S2–S4). Different lowercase letters denote statistically significant differences at *p* < 0.05. Data visualization was performed using the ggplot2 package [55].

Prior to multivariate analyses, all analyzed parameters were normalized using min–max scaling to a [0–1] range. Stress-indicative traits (TBARS, Proline, ΦNO, ΦNPQ, LTD, GSH, APX, CAT) were inverted such that higher normalized values consistently represented better physiological status, thereby ensuring interpretability across traits. A hierarchical cluster analysis was performed using the pheatmap package, employing Euclidean distance and complete linkage clustering [56].

## 5. Conclusions

This study demonstrates that urban environmental stressors have a significant impact on the structural, physiological, and biochemical traits of urban trees, with notable differences among species. By integrating multilevel trait data into a composite tree health risk index, we provide a comprehensive framework for evaluating species-specific strategies to withstand urban stress. Among the studied species, *Tilia* exhibited the highest sensitivity, characterized by substantial declines in photochemical efficiency, increased oxidative damage, and a marked decrease in THRI under urban conditions, particularly during the summer. *Celtis* exhibited moderate resilience, characterized by intermediate physiological responses and adaptive antioxidant activation. *Platanus* showed the most stable performance across structural, photosynthetic, and biochemical parameters, supported by relatively stable THRI values and minimal clustering differences between urban and control treatments, indicating robust tolerance to combined urban stressors.

These findings underscore the importance of species selection for urban greening and the utility of integrative indicators, such as chlorophyll fluorescence traits, oxidative stress biomarkers, and THRI, for early detection of stress and long-term health monitoring. *Platanus* emerged as the most promising candidate for sustainable urban planting due to its physiological stability, moderate biochemical response, and relatively stable health performances under environmental pressure. Overall, this study highlights the importance of utilizing a comprehensive set of plant functional traits to inform the selection of suitable species, guide practical urban greening decisions, and support the design of landscapes that can withstand climate-intensified urban stress.

## Figures and Tables

**Table 1:** Chlorophyll fluorescence and photosynthetic traits in leaves of *Tilia*, *Celtis,* and *Platanus* (mean ± SE, *n* = 12) exposed to control and urban environment during baseline (June) and summer stress peak (August). Results of the Two-way ANOVA indicate the significance of the timepoint (T), condition (C), and their interaction (TxC). Different letters within the same species and trait indicate significant differences according to Tukey’s HSD test (*p* < 0.05).

*Tilia*									
T	C	*Φ*_II_	*Φ*_NO_	*Φ*_NPQ_	*F*_v′_/*F*_m′_	*g*_H_^+^	*v* _H_^+^	LEF	*q*_L_
June	control	0.570 ± 0.03 ^a^	0.239 ± 0.01 ^a^	0.189 ± 0.04 ^c^	0.732 ± 0.02 ^a^	201.600 ± 19.3 ^a^	0.105 ± 0.01 ^a^	59.379 ± 2.34 ^a^	0.439 ± 0.02 ^bc^
	urban	0.488 ± 0.03 ^a^	0.226 ± 0.01 ^a^	0.278 ± 0.04 ^bc^	0.685 ± 0.02 ^a^	134.301 ± 11.73 ^b^	0.061 ± 0.01 ^ab^	20.493 ± 2.87 ^c^	0.505 ± 0.03 ^ab^
August	control	0.503 ± 0.04 ^a^	0.108 ± 0.01 ^b^	0.384 ± 0.03 ^ab^	0.698 ± 0.01 ^a^	135.982 ± 18.87 ^b^	0.032 ± 0.00 ^b^	48.250 ± 2.87 ^b^	0.575 ± 0.01 ^a^
	urban	0.298 ± 0.05 ^b^	0.215 ± 0.00 ^a^	0.497 ± 0.02 ^b^	0.432 ± 0.04 ^b^	56.018 ± 4.51 ^c^	0.068 ± 0.00 ^ab^	18.676 ± 1.43 ^c^	0.386 ± 0.02 ^c^
Two-way ANOVA	T	**	***	***	***	***	*	**	ns
	C	**	**	*	***	***	ns	***	*
	TxC	ns	***	ns	***	ns	**	*	***
***Celtis***									
T	C	*Φ*_II_	*Φ*_NO_	*Φ*_NPQ_	*F*_v′_/*F*_m′_	*g*_H_^+^	*v*_H_^+^	LEF	*q*_L_
June	control	0.581 ± 0.02 ^b^	0.263 ± 0.01 ^a^	0.155 ± 0.03 ^a^	0.753 ± 0.01 ^a^	224.308 ± 17.52 ^a^	0.047 ± 0.01 ^a^	39.086 ± 3.44 ^a^	0.482 ± 0.02 ^b^
	urban	0.646 ± 0.00 ^ab^	0.230 ± 0.00 ^ab^	0.123 ± 0.00 ^a^	0.759 ± 0.00 ^a^	155.828 ± 15.20 ^b^	0.061 ± 0.01 ^a^	26.269 ± 2.55 ^b^	0.566 ± 0.02 ^ab^
August	control	0.664 ± 0.00 ^a^	0.212 ± 0.00 ^b^	0.122 ± 0.00 ^a^	0.755 ± 0.00 ^a^	196.819 ± 13.86 ^ab^	0.055 ± 0.01 ^a^	19.810 ± 1.06 ^b^	0.648 ± 0.02 ^a^
	urban	0.615 ± 0.01 ^ab^	0.240 ± 0.01 ^ab^	0.143 ± 0.01 ^a^	0.753 ± 0.00 ^a^	141.261 + 12.41 ^b^	0.058 ± 0.00 ^a^	7.465 ± 0.83 ^c^	0.540 ± 0.03 ^b^
Two-way ANOVA	T	ns	*	ns	ns	ns	ns	***	*
	C	ns	ns	ns	ns	***	ns	***	ns
	TxC	**	**	ns	ns	ns	ns	ns	***
***Platanus***									
T	C	*Φ*_II_	*Φ*_NO_	*Φ*_NPQ_	*F*_v′_/*F*_m′_	*g*_H_^+^	*v*_H_^+^	LEF	*q*_L_
June	control	0.674 ± 0.01 ^a^	0.226 ± 0.00 ^a^	0.121 ± 0.00 ^b^	0.771 ± 0.00 ^a^	290.222 ± 23.94 ^a^	0.053 ± 0.01 ^a^	14.618 ± 0.97 ^ab^	0.623 ± 0.02 ^a^
	urban	0.642 ± 0.01 ^a^	0.225 ± 0.00 ^a^	0.126 ± 0.00 ^b^	0.754 ± 0.00 ^a^	234.226 ± 13.04 ^ab^	0.045 ± 0.00 ^a^	14.495 ± 0.67 ^ab^	0.594 ± 0.02 ^a^
August	control	0.586 ± 0.00 ^b^	0.189 ± 0.00 ^b^	0.233 ± 0.01 ^a^	0.711 ± 0.01 ^b^	176.104 ± 16.91 ^b^	0.046 ± 0.00 ^a^	15.595 ± 1.07 ^a^	0.573 ± 0.01 ^a^
	urban	0.527 ± 0.01^c^	0.221 ± 0.00 ^a^	0.269 ± 0.02 ^a^	0.652 ± 0.01 ^c^	83.324 ± 9.43 ^c^	0.040 ± 0.00 ^a^	11.319 ± 0.96 ^b^	0.589 ± 0.02 ^a^
Two-way ANOVA	T	***	**	***	***	***	ns	ns	ns
	C	**	*	ns	***	***	ns	*	ns
	TxC	ns	*	ns	*	ns	ns	*	ns

Significance codes: ns—not significant, * *p* < 0.05, ** *p* < 0.01, *** *p* < 0.001.

**Table 2:** Antioxidant and oxidative stress biomarkers in leaves of *Tilia*, *Celtis*, and *Platanus* exposed to control and urban environments during baseline (June) and peak summer stress (August). Data are presented as means ± standard error (SE) for TBARS (lipid peroxidation) [nmol mg^−1^ proteins], proline [nmol g^−1^ FW], glutathione (GSH) [µmol mg^−1^ protein], ascorbate peroxidase (APX) [U mg^−1^ protein], and catalase (CAT) [U mg^−1^ protein]. Two-way ANOVA indicates the significance of the timepoint (T), condition (C), and their interaction (TxC). Different letters within the same species and trait indicate statistically significant differences according to Tukey’s HSD test (*p* < 0.05).

*Tilia*						
T	C	TBARS	Proline	GSH	APX	CAT
June	control	4.02 ± 0.40 ^c^	108.83 ± 7.65 ^c^	7.06 ± 0.42 ^b^	7.86 ± 0.80 ^c^	0.70 ± 0.03 ^b^
	urban	6.75 ± 0.36 ^bc^	204.81 ± 23.13 ^b^	12.05 ± 1.53 ^b^	23.05 ± 1.42 ^b^	1.40 ± 0.17 ^b^
August	control	9.86 ± 1.03 ^b^	153.03 ± 1.35 ^b^	6.58 ± 0.65 ^b^	28.53 ± 1.93 ^b^	2.23 ± 0.09 ^b^
	urban	27.07 ± 2.00 ^a^	860.02 ± 11.11 ^a^	60.72 ± 2.88 ^a^	37.41 ± 1.51 ^a^	4.57 ± 0.78 ^a^
Two-way ANOVA	T	***	***	***	ns	ns
	C	***	***	***	***	**
	TxC	***	***	***	***	***
***Celtis***						
T	C	TBARS	Proline	GSH	APX	CAT
June	control	4.89 ± 0.30 ^bc^	29.82 ± 2.44 ^b^	11.82 ± 0.37 ^c^	15.48 ± 2.16 ^b^	4.72 ± 0.26 ^ab^
	urban	4.14 ± 0.35 ^c^	43.95 ± 1.02 ^b^	7.39 ± 1.10 ^c^	43.14 ± 7.92 ^a^	7.34 ± 2.07 ^a^
August	control	7.20 ± 0.79 ^b^	214.95 ± 10.38 ^a^	30.81 ± 3.24 ^b^	15.21 ± 0.91 ^b^	2.20 ± 0.30 ^b^
	urban	9.88 ± 0.64 ^a^	213.50 ± 16.82 ^a^	40.21 ± 2.00 ^a^	43.63 ± 7.92 ^a^	9.12 ± 0.25 ^a^
Two-way ANOVA	T	***	***	***	ns	ns
	C	ns	ns	ns	***	**
	TxC	*	ns	**	ns	ns
***Platanus***						
T	C	TBARS	Proline	GSH	APX	CAT
June	control	9.31 ± 1.08 ^c^	23.64 ± 4.14 ^c^	7.36 ± 0.78 ^b^	12.55 ± 1.00 ^b^	2.31 ± 0.71 ^ab^
	urban	13.31 ± 1.42 ^bc^	80.18 ± 2.07 ^b^	7.64 ± 0.37 ^b^	22.27 ± 0.95 ^b^	0.64 ± 0.10 ^b^
August	control	24.97 ± 1.56 ^b^	83.03 ± 3.33 ^b^	9.16 ± 0.24 ^ab^	14.36 ± 1.93 ^b^	3.38 ± 0.70 ^a^
	urban	42.89 ± 5.21 ^a^	111.36 ± 9.76 ^a^	12.73 ± 1.65 ^a^	57.96 ± 4.89 ^a^	1.96 ± 0.20 ^ab^
Two-way ANOVA	T	***	***	**	***	*
	C	**	*	ns	***	*
	TxC	*	***	ns	***	ns

Significance codes: ns—not significant, * *p* < 0.05, ** *p* < 0.01, *** *p* < 0.001.

**Table 3:** Overview of Photosynthesis- and Stress-Related Parameters.

Parameter	Full Name	Functional Significance	Reference
Structural and Water Status Indicators			
SPAD	Relative Chlorophyll Content Index	Indicates chlorophyll concentration and nitrogen status	[41]
LTD	Leaf Temperature Differential	Proxy for transpiration efficiency and water stress	[39]
LT	Leaf Thickness	Influences photosynthetic capacity and water retention	[39]
Chlorophyll Fluorescence Parameters (PSII Performance)			
*Ф*_II_	Quantum Yield of Photosystem II	Efficiency of light used for photochemical reactions.	[42]
*Ф*_NO_	Quantum Yield of Non-Regulated Energy Dissipation	Non-regulated energy loss, often linked to stress.	[39]
*Ф*_NPQ_	Quantum Yield of Regulated Non-Photochemical Quenching	Protective heat dissipation; excess energy regulation, toward reducing damage to plants	[39]
*F*_v′_/*F*_m′_	Ratio of Variable to Maximal Fluorescence (light-adapted)	Indicates PSII efficiency under actinic light	[42]
LEF	Linear Electron Flow	Total electron flow through PSII	[39]
*q*_L_	Redox status of PSII	fraction of PSII centers that are in the open state	[43]
ATP synthase activity and energy flux parameters linked to photophosphorylation			
*g*_H_^+^	Proton conductivity	Indicates ATP synthase activity and thylakoid proton flux	[44]
*v*_H_^+^	Proton flux	Correlates with ATP synthesis rate.	[45]

## Data Availability

The original contributions presented in this study are included in the article/Supplementary Material. Further inquiries can be directed to the corresponding author.

## Supplementary Materials

The following supporting information can be downloaded at: https://www.mdpi.com/article/10.3390/plants14223451/s1.
